# Do mturkers collude in interactive online experiments?

**DOI:** 10.3758/s13428-023-02220-3

**Published:** 2023-09-01

**Authors:** Razvan S. Ghita

**Affiliations:** Department of Business and Management, Southern Denmark University, Universitetsparken 1, Kolding, 6000 Denmark

**Keywords:** Experimental methodology, Behavioral research, Internet interactive experiments, Amazon mechanical turk, Collusion

## Abstract

One of the issues that can potentially affect the internal validity of interactive online experiments that recruit participants using crowdsourcing platforms is collusion: participants could act upon information shared through channels that are external to the experimental design. Using two experiments, I measure how prevalent collusion is among MTurk workers and whether collusion depends on experimental design choices. Despite having incentives to collude, I find no evidence that MTurk workers collude in the treatments that resembled the design of most other interactive online experiments. This suggests collusion is not a concern for data quality in typical interactive online experiments that recruit participants using crowdsourcing platforms. However, I find that approximately 3% of MTurk workers collude when the payoff of collusion is unusually high. Therefore, collusion should not be overlooked as a possible danger to data validity in interactive experiments that recruit participants using crowdsourcing platforms when participants have strong incentives to engage in such behavior.

## Introduction

Interactive online experiments that recruit participants using crowdsourcing platforms such as Amazon Mechanical Turk (MTurk) are growing in popularity (Amir, Rand, & Gal, 2012). Despite their benefits (Mason & Suri, 2012), interactive online experiments that recruit participants using crowdsourcing platforms impose a higher loss of experimental control compared to interactive laboratory experiments. One of the issues that might arise due to this loss of experimental control is collusion, i.e., participants could act upon information shared through channels that are external to the experimental design. If such collusion between participants is common, it could distort the interpretation of experimental results because most experimental designs assume no external communication between participants. For example, two MTurk workers (MTurkers) who are part of the same social network and perform similar types of tasks (Gray, Suri, Ali, & Kulkarni, 2016; Yin, Gray, Suri, & Vaughan, 2016), could communicate through a private messaging app while playing a coordination or public goods game (Arechar, Gachter, & Molleman, 2018; Guarin & Babin, 2021). Were such communication to exist, it should increase the level of cooperation (Cooper, DeJong, Forsythe, & Ross, 1992) which, in turn, might lead researchers to erroneously conclude that MTurkers have special characteristics that cause them to cooperate more than other groups of participants (Almaatouq, Krafft, Dunham, Rand, & Pentland, 2020). Additionally, collusion would introduce noise in experiments that involve information markets (Teschner & Gimpel, 2018) because it would allow participants to directly share (or lie about) their private information.

Although researchers have acknowledged collusion as a potential concern for data quality (Arechar, Gachter, & Molleman, 2018; Hawkins, 2015; Horton, Rand, & Zeckhauser, 2011), to my knowledge, no empirical evidence exists that quantifies how prevalent collusion is in interactive online experiments that recruit participants using crowdsourcing platforms. This study uses two experiments in which participants are incentivized to collude to provide such evidence about online participants recruited from MTurk. Furthermore, I investigate if collusion is affected by several design choices: the recruitment method, the instructions given to participants about communicating with others, the payoff of collusion, and the amount of time participants expect to wait between periods.

## When will MTurkers collude?

MTurkers will collude only if they have both the ability and the motivation to do so. On the one hand, evidence suggests that MTurkers have the ability to collude in interactive online experiments. First, MTurkers communicate on dedicated forums (Irani & Silberman, 2013). Although popular forums have policies against sharing specific information about MTurk tasks (human intelligence tasks, HITs), there could be lesser-known forums dedicated to sharing information about how to maximize payoffs in certain HITs (e.g., positioning and answers to attention checks, completion codes, and strategies for interactive online experiments). Second, some MTurkers are part of the same social network (Gray, Suri, Ali, & Kulkarni, 2016; Yin, Gray, Suri, & Vaughan, 2016). Moreover, some MTurkers likely live in the same household (Chandler, Mueller, & Paolacci, 2014). These MTurkers could directly communicate with each other either offline or using an instant messaging app. Indeed, Yin, Gray, Suri, and Vaughan (2016) find that 13.8% of MTurkers who are connected, communicate with each other using such one-on-one channels.

On the other hand, practical considerations indicate that MTurkers do not have the ability to collude. First, given that most HITs are not interactive online experiments (Hitlin, 2016), MTurkers might not have considered how to optimize their payoff in this relatively rare type of HIT. Therefore, MTurkers may not realize that collusion is profitable. Second, collusion partners may not have time to coordinate and join the same HITs because available spots in HITs are quickly filled by other MTurkers.

Ability is not sufficient for collusion to occur, MTurkers need to also be motivated to collude. Incentives are likely a critical factor in the motivation to collude. For collusion to occur, the expected payoff of collusion must exceed its expected cost. Therefore, the likelihood of collusion will depend on the experimental design, specifically, on how the design affects the probability of successful collusion and the payoff of collusion. For example, collusion should be more likely to occur in experiments in which the expected payoff of collusion is high such as experiments with high stakes (e.g., Raihani, Mace, & Lamba, 2013). Similarly, collusion should be more likely to occur when the experimental design involves large groups of participants (e.g., Suri & Watts, 2011) because potential colluders have a higher probability of being in the same group as their collusion partner.

Many MTurkers will likely understand that experimenters do not intend for them to share information with others during interactive online experiments and, as a result, will consider that collusion is cheating. Therefore, beyond the rational cost-benefit considerations of attempting collusion (Becker, 1968), behavioral factors will likely reduce MTurkers’ motivation to collude. Specifically, MTurkers’ motivation to attempt collusion might be decreased because they prefer to maintain a positive image as a non-cheater about themselves (Stets & Carter, 2012). Additionally, participant’s collusion motivation might be lowered by social controls such as internalized norms against cheating (Opp, 2013) and the fear that a potential collusion partner will judge them as a cheater (Nettle, Harper, Kidson, Stone, Penton-Voak, & Bateson, 2013). However, it is unclear whether these social controls will have a strong impact on MTurkers’ behavior because many MTurkers work from home and thus operate in a socially empty environment without direct observation or judgment from others (Kroher & Wolbring, 2015; Lindenberg, 2018).

The above discussion suggests that it is ex-ante unclear whether MTurkers will collude in interactive online experiments. It also suggests that collusion likely depends on experimental design choices. I therefore investigate whether four design choices affect collusion. First, the recruitment method might affect collusion. Researchers have suggested two strategies for recruiting participants from crowdsourcing platforms to participate in online interactive experiments (Arechar, Gachter, & Molleman, 2018; Mason & Suri, 2012). When using the standing panel method (Mason & Suri, 2012; Palan & Schitter, 2018), researchers first create a standing panel of participants by posting a short, lower-paying HIT in which MTurkers are only asked whether they would like to take part in an interactive experiment in the future. Afterward, researchers post the higher-payoff HIT for the experimental session and only allow participants who joined the standing panel to participate. When using the instant method (Arechar, Gachter, & Molleman, 2018), researchers recruit participants from all MTurkers who are available when the higher-payoff HIT for the experimental session is posted. I expect that recruiting participants through the standing panel method will result in more collusion compared to the instant method because potential colluders have a longer time window to coordinate and self-select into standing pools. Available spots are likely filled more slowly for the lower-payoff HITs through which participants sign up to the standing panel than for the higher-payoff HITs for the experimental session (Buhrmester, Talaifar, & Gosling, 2018).

Second, the instructions given to participants about communicating with each other might also impact collusion. Informing participants that communication with others is not allowed in the instructions might reduce collusion, as it clarifies that such behavior is considered cheating thus removing any moral ambiguity (Irlenbusch & Villeval, 2015). However, results from previous studies have been mixed with regard to such interventions. Consistent with the prediction that asking people not to communicate will decrease collusion, Goodman, Cryder, and Cheema (2013) find that asking MTurkers not to look up factual information decreases the percentage of correct answers given to a factual question, even when participants are incentivized to answer accurately. Inconsistent with this prediction, Bryan, Adams, and Monin (2013) find that asking participants not to cheat has no effect on cheating. Moreover, the intervention might even backfire and increase collusion because it could inform MTurkers who would have not otherwise considered collusion that communication with others is possible (Fosgaard, Hansen, & Piovesan, 2013). Overall, it is ex-ante unclear whether explicitly mentioning that communication with other participants is prohibited will affect collusion. I also examine if informing participants that communication is allowed affects information sharing. I expect that announcing that communication is allowed will increase information sharing because all participants will know that communication with other participants is possible and is not considered cheating (Abeler, Nosenzo, & Raymond, 2019).

Third, collusion likely depends on the payoff of successful collusion such that experiments with higher stakes (e.g., Raihani, Mace, & Lamba, 2013) will be more vulnerable to collusion. Even if MTurkers view collusion as cheating, the higher payoff of collusion will likely motivate some of them to ignore their moral concerns and to collude (Kajackaite & Gneezy, 2017). Moreover, a higher payoff will likely attract a different, more experienced sample of MTurkers (Hitlin, 2016) who may be more capable of colluding.

Fourth, collusion might depend on the amount of time MTurkers expect to wait between periods. There is likely variation in how quickly MTurkers expect to advance through experiments. For instance, in synchronous interactive experiments, larger groups may require more waiting between periods as participants need to wait for the slowest group member to finish a given period. A shorter expected waiting time between periods would likely increase the opportunity cost of engaging in collusion, as MTurkers could complete the study more quickly and move on to other HITs if they do not attempt collusion. In contrast, a longer expected waiting time between periods would lower the opportunity cost of collusion as it would not be possible to decrease the waiting time regardless of how quickly MTurkers finish a period. As a result, I hypothesize that a longer expected waiting time between periods will result in more collusion.

To examine these predictions, I conduct two experiments on MTurk. In the first experiment, I manipulate the recruitment method and the instructions given to participants about communication. Participants had the opportunity to earn $2.00 by colluding in this first experiment. To reduce the risk that MTurkers do not collude in Experiment 1 even though such behavior occurs in typical interactive experiments, I aimed to set the payoff for successful collusion that is slightly higher than what is typically used in interactive experiments. In the second experiment, I manipulated the time MTurkers expect to wait between periods. Moreover, I increased the incentives to collude such that, in the second experiment, participants could earn $25.00 by colluding. In the second experiment, I aimed to set the payoff for successful collusion that is representative of experiments where stakes are unusually high (e.g., Larney, Rotella, & Barclay, 2019).

## Experiment 1

### Method

Participants were recruited from MTurk and the experimental task was programmed in oTree (Chen, Schonger, & Wickens, 2016).^1^ To participate, MTurkers needed to reside in the United States, have completed at least 100 HITs, and have an approval rate of at least 90% on their previous HITs. The experiment could not be opened from a mobile device. I used the unique ID assigned to each MTurker to restrict participants from completing the study more than once. I collected data over three days and seventeen sessions. Each session collected data for only one of the six treatments. Participants had twenty minutes to complete the task after accepting the HIT.

The task description informed respondents that they would receive a base pay of $0.90 and a bonus of up to $2.00 for completing the task. After agreeing to the task, participants were redirected to a webpage where they could complete the task. Participants read the instructions and needed to correctly answer eight comprehension questions within two attempts to participate in the experiment and receive the participation fee. The instructions were still available to participants while answering the comprehension questions. Participants who did not correctly answer all the comprehension questions within two attempts were not allowed to participate in the experiment. Participants then completed the main task of the study. Participants did not receive any feedback about their performance while completing the task. On average, participants took 6.4 min to complete the task and received $1.01 for their work.

#### Number choosing task

For ten periods and in groups of six, participants chose a number from a list that contained ten numbers. Participants learned that, out of the ten numbers, nine numbers were unique to each participant and one number was common across all participants in their group. They earned a bonus of 20 cents for each period in which they and at least one other participant in their group chose the common number. If participants did not share information, they had a 10% chance of choosing the common number each period. If participants were to share information and compare their lists of numbers, they could choose the common number in each period. Therefore, participants had incentives to share information. The expected bonus for participants who did not share information was 8 cents^2^ while the expected payoff of successful information sharing was $2.00.

#### Recruitment method

Participants self-selected into one of two treatments: Standing and Instant. In the Standing treatment, I recruited participants from a pool of MTurkers who, before the experimental session, had indicated their willingness to participate in an interactive experiment. I posted a one-question HIT where participants indicated whether they wanted to be notified about an upcoming HIT in which they will interact with other MTurkers. The payoff for completing this HIT was 1 cent and 830 participants signed up to receive email notifications about the upcoming sessions. The notification emails were sent approximately one and a half hours before a session and informed participants about the time at which the session would begin. Following the recommendation of Mason and Suri (2012), I invited approximately three times the number of participants that I intended to recruit for a session. In the Instant treatment, I recruited participants from the pool of available MTurkers at the time of posting a session. MTurkers who joined the pool for the Standing treatment could not participate in the Instant sessions. As expected, participants had a longer time window to accept the lower-payoff HIT for signing up for the standing panel than they did for the higher-payoff HIT for the experimental session in the Instant treatment. The available spots for the standing panel HIT were never filled while the available spots for the experimental session HIT were filled immediately after being posted.

#### Instructions about communication

Participants were randomly allocated to three treatments: NoMentionC, ProhibitedC, and AllowedC. In the NoMentionC treatment, the instruction contained no explicit instructions about communication with other participants. The instructions in this treatment described the task as a "game of chance". This wording implied that information sharing was prohibited because the element of chance would disappear if participants could always choose the common number by comparing their lists of numbers. In the ProhibitedC treatment, the task was also described as a "game of chance" and the instructions indicated that "communication with other participants is strictly prohibited". In the AllowedC treatment, the task was only described as a "game" and the instructions indicated that "communication with other participants is allowed! This study does not provide a chat function. However, you are allowed to communicate with your group members through other methods such as forums". Data from this treatment were collected in the last sessions to avoid informing participants about the purpose of the study and creating a demand effect.

#### Measure

I define collusion as participants acting upon information shared through channels that are external to the experimental design. This definition does not include the situation in which an individual gains additional information by simultaneously performing a study multiple times because they have access to multiple MTurk accounts (Chandler, Mueller, & Paolacci, 2014). Information sharing, whether it occurs between distinct participants or between multiple instances of the experiment controlled by a single participant, results in respondents having information that the experimenter does not intend them to possess. However, it is important to distinguish between these two cases because they have different underlying causes, and, as a result, different mitigation strategies.

To measure collusion, I used a design inspired by the coin-flipping task (e.g., Abeler, Becker, & Falk, 2014). The variable of interest was the number of periods in which a participant chooses the common number. Participants had a 10% chance of choosing the common number each period if they did not share information with each other. Participants could choose the common number in each period if they shared information and compared their lists of numbers. This design allows me to detect whether participants are sharing information with each other by comparing the distribution of outcomes to the theoretical distribution absent information sharing. If participants did not share information, the outcomes should follow a binomial distribution. For each participant, the probability of choosing the common number exactly *k* times out of ten periods is given by the formula $Pr(k,10,0.1) = {10 \atopwithdelims ()k} \times p^k \times (1 - p)^{10 - k}$. In all treatments except for the AllowedC treatment, information sharing was prohibited. Consequently, I classify any information sharing between distinct participants in these treatments as collusion. In contrast, because participants were allowed to share information in the AllowedC treatment, information sharing became part of the experimental design. Therefore, in the AllowedC treatment, I measured information sharing during interactive experiments but not collusion.

Within each period, the common number was identical across all groups of each session.^3^ This design choice enabled participants to benefit from sharing information across different groups, unlike in most experiments where participants can only gain advantages by sharing information within their own group. By having a common number for all groups in a session, I can measure information sharing between participants who are in different groups. It is important to measure information sharing between participants who are in different groups even if, in most studies, sharing information with partners who are not in the same group is unprofitable. This is because participants who are not in the same group will likely add noise by experimenting with their choices to determine if they are in the same group as their partner.^4^

To gain a deeper understanding of participants’ motivations and strategies, I included at least one open-ended question in the post-experimental questionnaire in every treatment. In all treatments except AllowedC, participants were asked a single open-ended question about the strategy they used during the task. To prevent revealing the study’s purpose and creating a demand effect, I only asked specific questions about communication in the final sessions, i.e., in the AllowedC treatment where communication was explicitly allowed. In these sessions, participants were also asked to "explain what method you used to communicate with other participants if you tried to do so" and to "explain why you did not try to communicate with other participants if you chose not to do so".

### Data

In total, 650 MTurkers completed the experiment in January 2022. Participants were 52.46% male, 39.95% reported a bachelor’s degree as the highest education level obtained, and 51.08% reported having participated in at least 30 academic studies in the past month. The mean age was 40. Across all characteristics, participants in the ProhibitedC, NoMentionC, and AllowedC treatments were similar. Participants also did not differ in the measured characteristics between the three days of data collection. Participants who self-selected in the Standing and Instant treatments had different characteristics. Consistent with the idea that males self-select into higher-paying HITs (Litman, Robinson, Rosen, Rosenzweig, Waxman, & Bates, 2020; Litman & Robinson, 2020; Manzi, Rosen, Rosenzweig, Jaffe, Robinson, & Litman, 2021), the Standing treatment, which required MTurkers to first accept a 1-cent HIT, attracted fewer male participants (mean = 0.46, sd = 0.50) compared to the Instant treatment (mean = 0.58, sd = 0.49, difference = 0.12, t = 2.99, two-tailed *p* < 0.01). In addition, on the measure of risk aversion developed by Dohmen, Falk, Huffman, Sunde, Schupp, and Wagner (2011), participants reported being more risk-averse in the Standing treatment (mean = 4.95, sd = 2.58) than in the Instant treatment (mean = 5.88, sd = 2.65, difference = 0.93, t = 4.50, two-tailed *p* < 0.01).

### Results

**Fig. 1:**
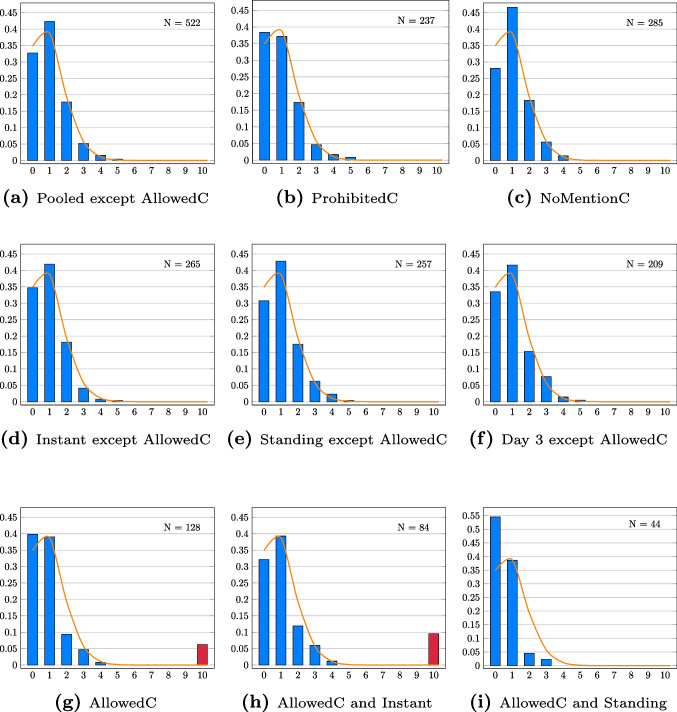
Empirical distribution of common number chosen by treatment in Experiment 1. Each figure provides the empirical distribution of common number chosen, along with the theoretical distribution absent information sharing across the treatments. The red bars highlight the density of respondents who chose the common number in all ten periods. The p-value for the $\chi ^2$ goodness of fit test of the null hypothesis that each distribution is similar to the theoretical distribution absent information sharing is >0.1 for all treatments except for the AllowedC treatment where it is < 0.01. This significant difference observed in the AllowedC treatment is driven by eight responses in which the common number was chosen in all ten periods. Further analysis of the open-ended questions suggests that these eight responses should be excluded from the analytical sample because they likely come from one individual with access to multiple MTurk accounts. Overall, the analyses suggest that collusion and information sharing between distinct individuals did not occur in any treatment

I first examine whether participants collude in the treatments that resemble most other experimental designs, i.e., all treatments in which participants are not explicitly told they are allowed to communicate. Table 1 and Fig. 1 show that the empirical distribution of the total number of periods in which participants chose the common number does not deviate significantly from the theoretical distribution absent information sharing (*p* = 0.80, $\chi ^2$ goodness of fit test; average probability of choosing the common number in a period is 10.15%, one-tailed *p* = 0.36, binomial test checking if the probability is greater than 10%) when pooling all the data except the observations from the AllowedC treatment. This suggests that, when conditions resemble most other experimental designs, MTurkers do not collude.

I find no evidence that the design choices studied affect the level of collusion when communication is not explicitly allowed. The distribution of the total number of periods in which participants chose the common in the ProhibitedC treatment is not significantly different from the one in the NoMentionC treatment (*p* = 0.13, two-tailed Kolmogorov-Smirnov (KS) test). Similarly, the distribution in the Instant treatment is not significantly different from that for the Standing treatment (*p* = 0.99, two-tailed KS test). Participants also did not collude more on the third day of data collection as compared to the first day (*p* = 0.99, one-tailed KS test) and second day (*p* = 0.82, one-tailed KS test).

Table 1 and Fig. 1 suggest that participants share information in the AllowedC treatment in which they are informed that communication with other participants is allowed. The empirical distribution of the total number of periods in which a participant chose the common number is significantly different from the theoretical distribution absent information sharing (*p* < 0.01, $\chi ^2$ goodness of fit test, probability of choosing the common number in a period is 13.75%, one-tailed *p* < 0.01, binomial test checking if the probability is greater than 10%). The effect is driven by eight observations in which the common number was chosen in all ten periods.^5^ It is unlikely that this pattern is generated by chance, given that the probability of choosing the common number in all ten periods by chance is $10^{-10}$.^6^^,^^7^

When further examining the eight responses in which the common number was chosen in all ten periods, I find that these responses are fraudulent and likely provided by the same individual who had access to multiple MTurk accounts. This is because all eight responses have nearly identical answers to the three open-ended questions. All eight responses are "I just guessed the number" to the question that asked how participants chose the numbers. Similarly, the responses are "I didn’t communicate with other participants" to the question that asked to explain what method participants used to communicate. Finally, the responses are "I don’t have option" to the questions that asked to explain why they did not communicate with other participants. The only difference between the answers is that some participants include an apostrophe in "don’t" while others do not. Because standard research protocol does not allow multiple responses from the same individual within the same study, I do not classify these responses as instances of information sharing between distinct participants. Instead, similar to previous studies (Bentley, Bloomfield, Bloomfield, & Lambert, 2023; Goodrich, Fenton, Penn, Bovay, & Mountain, 2023; Griffin, Martino, LoSchiavo, Comer-Carruthers, Krause, Stults ... & Halkitis, 2021), I classify these responses as coming from fraudulent accounts based on the nearly identical answers to the open-ended questions. I therefore removed these responses from the dataset used to make inferences about information sharing between distinct participants and collusion.

When excluding these eight responses, I find that the distribution of the total number of periods in which a participant chose the common number is not significantly different from the theoretical distribution absent information sharing in the AllowedC treatment (*p* = 0.62, $\chi ^2$ goodness of fit test). Overall, the analyses suggest that collusion and information sharing between distinct individuals do not occur in any treatment.

To provide recommendations for identifying fraudulent accounts, I further analyze the eight responses that are most likely coming from such fraudulent accounts. None of these responses are from the Standing treatment, which suggests that this recruitment method may help reduce fraudulent responses.^8^ The eight responses come from different IP addresses. Data from https://iphub.info indicates that five (62.50%) out of the eight responses came from a VPN as compared to 98 (15.26%) out of the 642 participants in the rest of the sample.

**Table 1:** Common numbers chosen by treatment in Experiment 1

Treatment	Pooled	AllowedC	ProhibitedC	NoMentionC	Instant	Standing	Theor.
Common numbers chosen	except				except	except	prob.
	AllowedC				AllowedC	AllowedC	
0	171 (32.76%)	51 (39.84%)	91 (38.40%)	80 (28.07%)	92 (34.72%)	79 (30.74%)	34.87%
1	221 (42.34%)	50 (39.06%)	88 (37.13%)	133 (46.67%)	111 (41.89%)	110 (42.80%)	38.74%
2	93 (17.82%)	12 (9.38%)	41 (17.30%)	52 (18.25%)	48 (18.11%)	45 (17.51%)	19.37%
3	27 (5.17%)	6 (4.69%)	11 (4.64%)	16 (5.61%)	11 (4.15%)	16 (6.23%)	5.74%
4	8 (1.53%)	1 (0.78%)	4 (1.69%)	4 (1.40%)	2 (0.75%)	6 (2.33%)	1.12%
5	2 (0.38%)	0 (0.00%)	2 (0.84%)	0 (0.00%)	1 (0.38%)	1 (0.39%)	0.01%
6	0 (0.00%)	0 (0.00%)	0 (0.00%)	0 (0.00%)	0 (0.00%)	0 (0.00%)	<0.01%
7	0 (0.00%)	0 (0.00%)	0 (0.00%)	0 (0.00%)	0 (0.00%)	0 (0.00%)	<0.01%
8	0 (0.00%)	0 (0.00%)	0 (0.00%)	0 (0.00%)	0 (0.00%)	0 (0.00%)	<0.01%
9	0 (0.00%)	0 (0.00%)	0 (0.00%)	0 (0.00%)	0 (0.00%)	0 (0.00%)	<0.01%
10	0 (0.00%)	8 (6.25%)	0 (0.00%)	0 (0.00%)	0 (0.00%)	0 (0.00%)	<0.01%
N	522	128	237	285	265	257	
p-values - $\chi ^2$ tests	0.80	<0.01	0.40	0.51	0.97	0.69	
p-values - Binomial tests							
0	0.85	0.14	0.14	0.99	0.54	0.93	
1	0.05	0.50	0.72	<0.01	0.16	0.10	
2	0.83	0.99	0.81	0.71	0.72	0.80	
3	0.74	0.75	0.80	0.57	0.90	0.41	
4	0.23	0.76	0.27	0.39	0.80	0.07	
5	0.18	1.00	0.05	1.00	0.33	0.32	
6	1.00	1.00	1.00	1.00	1.00	1.00	
7	1.00	1.00	1.00	1.00	1.00	1.00	
8	1.00	1.00	1.00	1.00	1.00	1.00	
9	1.00	1.00	1.00	1.00	1.00	1.00	
10	1.00	<0.01	1.00	1.00	1.00	1.00	
Prob. - common number	10.15%	13.75%	9.66%	10.56%	9.55%	10.78%	10.00%

The table reports the empirical distribution of the number of periods in which participants chose the common number in all treatments in Experiment 1. The last column reports the theoretical probability of choosing the common number for a given number of periods absent information sharing. The table also reports the p-value for $\chi ^2$ goodness of fit test that checks whether the empirical distribution of the number of periods in which participants chose the common number in all treatments is different from the theoretical distribution absent information sharing. Next, the table reports one-tailed binomial tests that check if there are more participants who chose the common number for a specific number of periods than would be predicted if participants do not share information in all treatments. Finally, the table presents the probability of choosing the common number in one period in all treatments

Finally, I use the answers to the open-ended question in the AllowedC treatment that asked participants why they did not try to communicate with other participants to investigate why most participants did not attempt to share information. I classify these answers into three categories: no ability to communicate with other participants, insufficient time, and insufficient incentives. I can classify 85 out of 128 answers (66.41%) into at least one of these three categories. Most participants, 63 out of 128 (49.22%) indicate that they did not have the ability to communicate with other participants. Another group of participants, 18 out of 128 (14.06%), indicate that the incentives were not high enough for them to attempt to communicate. Finally, 16 out of 128 (12.5%) participants indicate that the twenty minutes they had available to complete the HIT were insufficient to successfully coordinate with other participants. Furthermore, 13 out of 128 (10.16%) participants indicate they attempted to share information through forums but could not find any posts about the task. I also checked all the MTurk forums that I am aware of (Reddit, MTurkcrowd, Turkerview, MTurkforum) and could not find specific information about the task. This indicates that information sharing was not attempted between strangers who communicated through forums.

### Discussion

In the treatments that resemble the design of most other interactive online experiments that recruit participants using crowdsourcing platforms, I find no evidence of collusion. This suggests that MTurkers will not collude in the majority of interactive online experiments. However, my results suggest that eight fraudulent accounts, likely controlled by a single individual, shared information between different instances of the experiment. Thus, another cost of not filtering out fraudulent respondents from studies is that these participants might be more likely to collude. Previous research provides extensive recommendations for how to filter out such fraudulent responses (e.g., Aguinis, Villamor, & Ramani, 2021; Burnette, Luzier, Bennett, Weisenmuller, Kerr, Martin... & Calderwood, 2022; Chandler, Sisso, & Shapiro, 2020; Griffin, Martino, LoSchiavo, Comer-Carruthers, Krause, Stults... & Halkitis, 2021; Moss & Litman, 2018; Yarrish, Groshon, Mitchell, Appelbaum, Klock, Winternitz, & Friedman-Wheeler, 2019). In my sample, the eight fraudulent responses came mostly from accounts that used a VPN and these responses were exclusively observed in the Instant treatment. Thus, it is possible that blocking VPN usage (see also Chandler, Sisso, and Shapiro, 2020; Dennis, Goodson, & Pearson, 2020; Kennedy, Clifford, Burleigh, Waggoner, Jewell, & Winter, 2020) and recruiting respondents via standing panels can partially mitigate against MTurkers who hold multiple accounts. It should be noted, however, that such mitigation measures also have drawbacks. Specifically, both methods can block legitimate respondents from participating and thus limit the available sample. Moreover, recruiting MTurkers using the standing panel method can affect the demographics of the available pool and slow data collection speed. Finally, the analysis of the open-ended responses suggests that it is logistically difficult for MTurkers to communicate with each other during studies. Indeed, around half of the participants indicated that they did not have the ability to communicate with others. For the MTurkers who reported the ability to communicate, the practical challenges associated with communication made it unprofitable for them to make such attempts. This suggests that, even though channels for communication exist, the logistical difficulty of communicating with others prevents most MTurkers from colluding. Some MTurkers indicated that the maximum possible bonus of $2.00 was too low to warrant the effort to communicate. Thus, it is possible that MTurkers might collude in experiments in which the payoff of collusion is higher. To test this possibility, I increase the bonus that participants can earn from colluding in Experiment 2.

## Experiment 2

### Method

Experiment 2 employed the same design and measures as Experiment 1, with four exceptions. First, the incentives to collude were higher. Participants could earn a maximum bonus of $25.00^9^ (a bonus of $2.50 for each period in which they and a member of their group chose the common number) in Experiment 2 as compared to $2.00 in Experiment 1. Second, Experiment 2 manipulated the time MTurkers expect to wait between periods. Participants were assigned to either the Wait treatment, which required a 60-second pause between periods, or to the NoWait treatment, where they could progress through the experiment without any imposed waiting time between periods.^10^ Third, the base pay increased to $3.00 in Experiment 2 as compared to $0.90 in Experiment 1. Fourth, participants had 50 min to complete the task after accepting the HIT as compared to twenty minutes in Experiment 1. The latter two changes aimed to provide fair compensation and enough time to complete the study for participants in the Wait treatment. Experiment 2 kept constant communication about collusion by not mentioning communication (similar to the NoMentionC treatment from Experiment 1) and the recruitment method by recruiting all participants using the instant method. Similar to the NoMentionC treatment from Experiment 1, I only included one open-ended question in the post-experimental questionnaire, in which participants were asked to explain their strategy on the number-choosing task. I did not explicitly ask participants about communication to avoid revealing the purpose of the study and creating a demand effect.

On average, participants took 8.0 min (19.9 min) to complete the task and received $4.30 ($5.08) for their work in the NoWait (Wait) treatment. Participants who opened the link that allowed them to start Experiment 1 could not participate in Experiment 2.

### Data

In total, 320 MTurkers completed the experiment in November 2022. Participants were 49.06% male, 88.75% reported a bachelor’s degree as the highest education level obtained, and 23.13% reported having participated in at least 30 academic studies in the past month. The mean age was 35.^11^ Across all characteristics, participants in the Wait and NoWait treatments were similar.

### Results

**Fig. 2:**
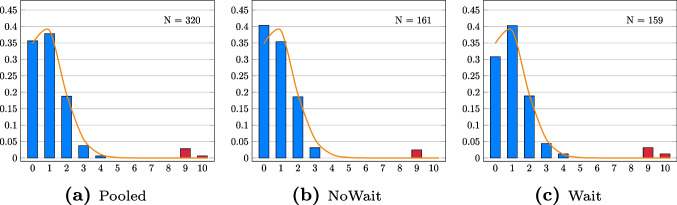
Empirical distribution of common number chosen by treatment in Experiment 2. Each figure provides the empirical distribution of common number chosen, along with the theoretical distribution absent information sharing across the treatments. The red bars highlight the density of respondents who chose the common number in at least nine periods. The p-value for the $\chi ^2$ goodness of fit test of the null hypothesis that each distribution is similar to the theoretical distribution absent information sharing is < 0.01 for both treatments

Table 2 and Fig. 2 show that the empirical distribution of the total number of periods in which participants chose the common number deviates significantly from the theoretical distribution absent information sharing when pooling all the data (*p* < 0.01, $\chi ^2$ goodness of fit test) and when examining each treatment individually (*p* < 0.01, $\chi ^2$ goodness of fit test in the Wait and NoWait treatments). The effects are driven by nine participants who chose the common number in nine periods and by two participants who chose the common number in all ten periods (*p* = 0.97, $\chi ^2$ goodness of fit test, if these participants are excluded). All of these eleven participants have likely colluded, given that the probability of choosing the common number in at least nine periods by chance is lower than $10^{-8}$. These results suggest that in experiments with unusually high payoffs from collusion, a small subset of MTurkers (3.43% in this case) collude.

**Table 2:** Common numbers chosen by treatment in Experiment 2

Treatment	Pooled	NoWait	Wait	Theor.
Common numbers chosen				prob
0	114 (35.63%)	65 (40.37%)	49 (30.82%)	34.87%
1	121 (37.81%)	57 (35.40%)	64 (40.25%)	38.74%
2	60 (18.75%)	30 (18.63%)	30 (18.87%)	19.37%
3	12 (3.75%)	5 (3.11%)	7 (4.40%)	5.74%
4	2 (0.63%)	0 (0.00%)	2 (1.26%)	1.12%
5	0 (0.00%)	0 (0.00%)	0 (0.00%)	0.01%
6	0 (0.00%)	0 (0.00%)	0 (0.00%)	<0.01%
7	0 (0.00%)	0 (0.00%)	0 (0.00%)	<0.01%
8	0 (0.00%)	0 (0.00%)	0 (0.00%)	<0.01%
9	9 (2.81%)	4 (2.48%)	5 (3.14%)	<0.01%
10	2 (0.63%)	0 (0.00%)	2 (1.26%)	<0.01%
N	320	161	159	
p-values - $\chi ^2$ tests	<0.01	<0.01	<0.01	
Prob. - common number	12.06%	10.43%	13.71%	10.00%

The table reports the empirical distribution of the number of periods in which participants chose the common number in all treatments in Experiment 2. The last column reports the theoretical probability of choosing the common number for a given number of periods absent information sharing. The table also reports the p-value for $\chi ^2$ goodness of fit test that checks whether the empirical distribution of the number of periods in which participants chose the common number in all treatments is different from the theoretical distribution absent information sharing. Finally, the table presents the probability of choosing the common number in one period in all treatments

Although more participants collude in the Wait treatment (seven participants) compared to the NoWait treatment (four participants), the distribution of the total number of periods in which participants chose the common in the Wait treatment is not significantly different from the one in the NoWait treatment (*p* = 0.23, one-tailed KS test). The probability that participants chose the common number in a given period is higher (13.71%) in the Wait than in the NoWait treatment (10.43%, logit coeff. 0.16, z = 2.84, one-tailed *p* < 0.01). However, this result is partially driven by the fact that participants in the Wait treatment are less likely to choose the common number in zero periods compared to participants in the NoWait treatment. This difference in the probability of choosing the common number in exactly zero periods is likely caused by noise instead of collusion. The difference in probability of choosing the common number between the Wait and NoWait treatments becomes only marginally significant (logit coeff. 0.15, z = 1.34, one-tailed *p* = 0.09) when dropping the participants who chose the common number in zero periods. Thus, the results provide weak evidence that a higher expected wait time between periods will increase collusion. Given that, as explained in Footnote 10, my manipulation of a higher expected wait time between periods is unusually strong, I do not interpret these results as suggesting that researchers should be more concerned about collusion when their experiment requires a longer waiting time between periods.

Similarly to the first experiment, the eleven participants who colluded have different IP addresses from one another and none of the participants mention information sharing as a strategy in the post-experimental questionnaire. Differently from the first experiment, colluding participants do not give nearly identical answers to the open-ended question related to the strategy they used and most of them do not use a VPN. Data from https://iphub.info indicates that one (9.09%) out of the eleven participants was using a VPN as compared to 18 (5.83%) out of the 309 participants in the rest of the sample. This suggests that collecting data from participants who do not use a VPN has little to no impact on the likelihood of collusion in high-stakes experiments.

## Conclusion

I investigate whether participants collude in interactive online experiments that recruit participants using crowdsourcing platforms. I collected data from 970 MTurkers through two incentivized experiments that allowed me to detect collusion if it occurs. Additionally, I examine how four design choices influence collusion: the recruitment method, the instructions given to participants about communicating with others, the payoff of collusion, and the amount of time participants expect to wait between periods.

This study is subject to limitations that provide opportunities for future research. First, the conclusion drawn from Experiment 1 that MTurkers do not collude in typical interactive online experiments hinges on the assumption that the payoff for collusion in Experiment 1 is higher than it is in typical experimental designs. I aimed to set a payoff of successful collusion in Experiment 1 that is higher than typical experimental designs to reduce the risk that MTurkers do not collude in Experiment 1 even though such behavior occurs in typical interactive experiments. Nonetheless, estimating the typical payoff for successful collusion is challenging due to the variation in MTurkers’ earnings per hour (Hitlin, 2016; Moss, Rosenzweig, Robinson, Jaffe, & Litman, 2020) and per academic study (Brodeur, Cook, & Heyes, 2022). As a result, it is possible that many experimental designs will provide a payoff for collusion that is higher than the one I have set in Experiment 1. In such designs, researchers should consider the strength of participants’ incentives for collusion before dismissing concerns about collusion. Second, beyond excluding fraudulent accounts from participating in studies, which most researchers likely already aim to do, I did not find any way to mitigate collusion. This leaves open-ended questions about what mitigation methods should be used in studies that are particularly vulnerable to collusion. Third, my experimental design cannot definitively identify whether information sharing occurred between distinct people, or whether a single individual was utilizing multiple MTurk accounts to share information across different instances of the experiment. Although information sharing reduces data quality regardless of how it is implemented, it would likely be more worrying to know that one person has access to multiple MTurk accounts. People with multiple MTurk accounts would likely also decrease the data quality of non-interactive experiments because they would observe multiple treatments. Therefore, future research could investigate how common it is for people to have access to multiple MTurk accounts.

Notwithstanding these limitations, my study contributes to the growing literature that investigates the advantages and disadvantages of using online labor markets as a method to recruit participants in interactive experiments (Arechar, Gachter, & Molleman, 2018; Hawkins, 2015; Horton, Rand, & Zeckhauser, 2011; Mason & Suri, 2012; Raihani, Mace, & Lamba, 2013; Thomas & Clifford, 2017). My findings suggest that in the vast majority of interactive online experiments that recruit participants through crowdsourcing platforms, collusion is not a significant concern. However, I also found that fraudulent accounts, likely controlled by one individual, shared information across multiple instances of the experiment. This suggests that information sharing is an additional risk to data quality that arises when researchers do not filter out fraudulent responses from their sample (Chandler, Sisso, & Shapiro, 2020). In addition to the extensive recommendations already provided by previous studies (e.g., Aguinis, Villamor, & Ramani, 2021), my results tentatively suggest that researchers should also consider recruiting participants using a standing panel (Mason & Suri, 2012; Palan & Schitter, 2018) and not allowing MTurkers who use a VPN to join the study (Kennedy, Clifford, Burleigh, Waggoner, Jewell, & Winter, 2020) to reduce participation from fraudulent accounts.

I also find that approximately 3% of MTurkers collude when the payoff for collusion is unusually high. Therefore, collusion should not be overlooked as a possible danger to data validity in experiments where participants have strong incentives to collude, for example, experiments in which the stakes are unusually high (Exley, 2018; Farjam, Nikolaychuk, & Bravo, 2019; Keuschnigg, Bader, & Bracher, 2016; Larney, Rotella, & Barclay, 2019; Raihani, Mace, & Lamba, 2013; Wu, Balliet, & Van Lange, 2016) or that form large groups of participants (Balietti & Riedl, 2021; Faravelli, Kalayci, & Pimienta, 2020; Suri & Watts, 2011). Despite the unusually high payoff in Experiment 2, I observed relatively low levels of collusion. This, coupled with participant responses to open-ended questions from Experiment 1, suggests that logistical challenges impede most MTurkers from colluding during interactive online experiments, even though channels for communication are available.

## Footnotes

1 The oTree code can be downloaded at https://doi.org/10.6084/m9.figshare.23641722. The instructions can be found at https://doi.org/10.6084/m9.figshare.19940987.

2 The probability of choosing the common number was 0.1 in each period. To win the bonus in a period in which a participant chose the common number, another participant from the group needed to also choose the common number. With five other participants in the group, the probability of all of them not choosing the common number was 0.59 ($0.9^5$). Thus, the probability of at least one group member choosing the common number was 0.41 ($1 - 0.59$) each period. Therefore, the probability of winning the bonus in a period was 0.04 ($0.41 \times 0.1$). As a result, for all ten periods, a participant was expected to win 0.4 ($0.04 \times 10$) times corresponding to an expected payoff of 8 cents ($0.4 \times 20$ cents).

3 Participants were informed that the number was common across their group but were not explicitly informed that the number was also common across all groups in a session.

4 To decrease waiting time, participants did not need to wait for all other participants in their group to reach the same completion level to advance in the experiment. Therefore, some groups contained fewer than six participants because participants did not wait for groups of six to form before beginning the task and because some MTurkers accepted the HIT but did not participate in the study. As a result, the number of participants in some treatments is not a multiple of six. I simulated additional participants after the experiment so that all participants had incentives as if they were part of groups of six. Participants who were part of smaller groups were therefore not disadvantaged because participants received no feedback during the experiment and because the common number was identical across all groups of each session.

5 Out of the eight responses, three responses were in the same group. Therefore, if respondents could only share information with their group members, then three out of these eight respondents would have increased their payoff by sharing information.

6 In the other treatments, colluding participants could have intentionally not chosen the common number in all periods to avoid detection. If colluding participants used this strategy, I should observe more participants than expected choosing the common number for a number of periods that is profitable but that makes it difficult to know which participant colluded (e.g., three to five times). I find no evidence of participants using such a strategy. Two participants chose the common number five times although the probability of doing so was 0.01%. However, when examining the amount of time spent by these participants on the task, I find no evidence of collusion. On average, these two participants spent less time on each period of the task (4.7 s) than participants who chose the common number in all ten periods (38.1 s) and they spent less time in periods in which they chose the common number (3.9 s) than in periods in which they did not choose the common number (5.4 s).

7 The observed pattern cannot be explained by these participants using a semi-automated script (Buchanan, & Scofield, 2018) in the number-choosing task because each participant saw a different list of nine numbers unique to them. Therefore, any rule used by a script such as choosing the lowest/highest number cannot explain participants choosing the common number in all ten periods.

8 An analysis of the entire sample suggests a similar conclusion. I find that 20 out of 650 respondents gave an answer that was unusual and similar to at least one other respondent (e.g., "based on calculation" and "just follow the instructions") to the question that asked how they chose the numbers. Only 2 out of these 20 unusual responses are from the Standing treatment.

9 Van Zant, Kennedy, and Kray (2022) ask MTurkers what they consider to be a large maximum bonus and find that, on average, MTurkers consider a large bonus to be $12.31. Therefore, most MTurkers will consider $25.00 to be a large maximum bonus.

10 The manipulation of expected waiting time is unusually strong because there are likely few interactive online experiments in which MTurkers expect to wait such a long time, 60 s, between periods. An alternative, more realistic manipulation would have imposed that participants wait for the slowest member of the group before proceeding. However, this more realistic manipulation would have had a higher risk that my experiment could not detect that a higher expected wait time increases collusion even though this relationship exists in reality. I opted for a strong manipulation to reduce this risk.

11 Interestingly, participants who self-selected in this experiment with higher stakes were more educated, younger, and reported participating in fewer academic studies than participants who self-selected in the first, lower-stakes experiment.

## Declarations

### Open Practices Statement

The datasets generated during and/or analysed during the current study are available in the Figshare repository, (https://doi.org/10.6084/m9.figshare.19936070). The oTree code can be downloaded at (https://doi.org/10.6084/m9.figshare.23641722). The experimental designs were preregistered (https://aspredicted.org/a89wc.pdf for Experiment 1 and https://aspredicted.org/rq2qg.pdf for Experiment 2). Only the main analyses were preregistered. All other analyses were exploratory
